# Phylogeographic evidence of cognate recognition site patterns and transformation efficiency differences in *H. pylori*: theory of strain dominance

**DOI:** 10.1186/1471-2180-13-211

**Published:** 2013-09-19

**Authors:** Ana Maldonado-Contreras, Shrinivasrao P Mane, Xue-Song Zhang, Luis Pericchi, Teresa Alarcón, Monica Contreras, Bodo Linz, Martin J Blaser, María Gloria Domínguez-Bello

**Affiliations:** 1 Department of Biology, University of Puerto Rico, Río Piedras, San Juan, PR, USA; 2 Microbiology and Physiological Systems Department, University of Massachusetts Medical School, Worcester, MA, USA; 3 Virginia Bioinformatics Institute, Virginia Tech, Blacksburg, VA, USA; 4 Department of Medicine, New York University Langone Medical Center, Manhattan, NY, USA; 5 Department of Mathematic, University of Puerto Rico, Río Piedras, San Juan, USA; 6 Servicio de Microbiología, Hospital Universitario de la Princesa, Madrid, Spain; 7 Venezuelan Institute of Scientific Research (IVIC), San Antonio de los Altos, Venezuela; 8 Department of Biochemistry and Molecular Biology, Pennsylvania State University, University Park, PA, USA; 9 New York Harbor Veterans Affairs Medical Center, Manhattan, NY, USA

**Keywords:** *H. pylori*, Haplotypes, Restriction-Modification system, Recombination

## Abstract

**Background:**

*Helicobacter pylori* has diverged in parallel to its human host, leading to distinct phylogeographic populations. Recent evidence suggests that in the current human mixing in Latin America, European *H. pylori* (hpEurope) are increasingly dominant at the expense of Amerindian haplotypes (hspAmerind). This phenomenon might occur via DNA recombination, modulated by restriction-modification systems (RMS), in which differences in cognate recognition sites (CRS) and in active methylases will determine direction and frequency of gene flow. We hypothesized that genomes from hspAmerind strains that evolved from a small founder population have lost CRS for RMS and active methylases, promoting hpEurope’s DNA invasion. We determined the observed and expected frequencies of CRS for RMS in DNA from 7 *H. pylori* whole genomes and 110 multilocus sequences. We also measured the number of active methylases by resistance to *in vitro* digestion by 16 restriction enzymes of genomic DNA from 9 hpEurope and 9 hspAmerind strains, and determined the direction of DNA uptake in co-culture experiments of hspAmerind and hpEurope strains.

**Results:**

Most of the CRS were underrepresented with consistency between whole genomes and multilocus sequences. Although neither the frequency of CRS nor the number of active methylases differ among the bacterial populations (average 8.6 ± 2.6), hspAmerind strains had a restriction profile distinct from that in hpEurope strains, with 15 recognition sites accounting for the differences. Amerindians strains also exhibited higher transformation rates than European strains, and were more susceptible to be subverted by larger DNA hpEurope-fragments than *vice versa*.

**Conclusions:**

The geographical variation in the pattern of CRS provides evidence for ancestral differences in RMS representation and function, and the transformation findings support the hypothesis of Europeanization of the Amerindian strains in Latin America via DNA recombination.

## Background

*H. pylori* has accompanied humans throughout evolution [1], and as humans diverged, so did *H. pylori.* Based on multilocus sequences (MLS), *H. pylori* strains can be divided into populations that are specific for the geographic origin of their human hosts [1-4]. Strains from present-day Africans include the most ancestral population hpAfrica2 from Southern Africa, hpNEAfrica from northeastern Africa and hpAfrica1 from western (sub-population hspWAfrica) and southern Africa (hspSAfrica). *H. pylori* from Europe, the Middle East, western Asia and India belong to the hpEurope population, and strains from Asians include hpAsia2 and hpEastAsia. The latter is subdivided into hspEAsia (from East Asians), hspAmerind (from Native Americans), and hspMaori (from Pacific islanders). About 80% of the *H. pylori* strains isolated from Mestizo hosts in Latin America were assigned to hpEurope and almost 20% to hspWAfrica, but no strains were assigned to hspAmerind [5]. Conversely, *H. pylori* strains isolated from Latin America Amerindian hosts showed multi-locus haplotypes of the hspAmerind and hpEurope populations in relatively equal proportions [2,5].

Geographic clustering also has been shown in virulence-associated genes, such as *vacA*[6-8]. All *H. pylori* strains recovered to date from Mestizo hosts have carried European-types (*s2, s1a, s1b*) of *vacA*, while the ones recovered from Amerindian hosts exhibited similar amounts of *vacA* subtype *s1c -*clustering with East Asia-Pacific isolates- and European *vacA* subtype *s1a* and *s1b*[9].

We have also shown that the hpEurope strains isolated from Mestizos and Amerindians in Latin America hosts exhibit a mosaic genetic structure; they are of predominantly European ancestry, containing some introgressions from African or Asian strains [5]. Thus, this mosaicism suggests Europeanization of the DNA from the original Latin America Amerindian strains through several events of recombination [10], including transformation [11] or conjugation-like mechanisms [12]. *H. pylori* population dynamics is known to be shaped by DNA transformation and recombination, and the recombination rate in this bacterium is extraordinarily high [11,13]. Since several genetically distinct *H. pylori* strains can co-colonize a single stomach [9,14,15] and since *H. pylori* are highly competent [16,17], the net direction of transformation determines which genome would be invaded by foreign DNA [18]. Instead of replacement of less fit strains, allelic competition via recombination among strains seems to dominate *H. pylori* evolution [19-21]. Recombination, as evidenced by the mosaic genetic structure of strains recovered from Mestizo and European hosts, suggests the co-existence of at least two different haplotype-strains in a single host [14] that allows recombination and provides a mechanism of competition, in this case, allelic competition rather than strain competition.

Bacterial restriction-modification systems (RMS) confer protection against invasion by foreign DNA, for example that from bacteriophages [22], or from other bacteria [18], by cleavage of this foreign DNA. In general, RMS consist of a restriction endonuclease (RE) that recognizes and cleaves specific DNA sequences (cognate recognition sites), and a counterpart methylase that catalyses the addition of a methyl group to adenine or cytosine residues in the same cognate recognition sites, protecting it from restriction by the cognate enzyme [23]. According to their subunit composition, cofactor requirements, such as ATP, AdoMet, or/and Mg^+2^ and mode of action, RMS can be divided into types I, II, IIS, and III. Type II RMSs are the simplest and most widely distributed among *H. pylori* strains [24,25], in which methylases and restriction enzymes act independently. Type II cognate recognition sites are often palindromic, 4–8 nt in length, with continuous (i.e. GATC) or interrupted (i.e. GCCNNNNNGGC) palindromes [26]. Similarly, Type IIS RMSs, also found in *H. pylori*, have independent restriction and methylation enzymes, but the endonucleases act as monomers, restriction sites are uninterrupted (4-7nt), and DNA cleavage occurs at specific distances from the recognition sites.

When cognate recognition sites are frequent, genomic or plasmid DNA can be extensively cut, impairing recombination [27]. However, cognate recognition sites also play a role in recombination, since they provide the locus for double stranded cuts suitable as substrate for recombination. Therefore, depending on the relative frequency of the cognate recognition sites, DNA restriction and methylation systems modulate the capability of DNA to recombine. As such, we hypothesized that the dominance of hpEurope strains in Latin America might be due to differences in the cognate restriction sites and active methylases between Amerindian and European strains. To test this hypothesis, we studied the frequencies of cognate recognition sites for 32 restriction enzymes in *H. pylori* strains that were assigned to different populations. In addition, we estimated the number of active methylases and compared transformation rates in hpEurope and hspAmerind *H. pylori* strains. Thus, we provide evidence of specific recombination events and mechanisms that indicate preferential receptor and donor status, respectively, in Amerindian and European strains.

## Results

### Observed and expected number of cognate recognition sites

We examined the published multi-locus sequences (MLS) of 110 *H. pylori* strains (Additional file 1: Figure S1 and Table 1) [2,10]. The previously assigned MLS-based haplotypes were consistent with the geographic origin of their hosts: all of the *H. pylori* sequences from strains from European hosts were assigned to hpEurope [2,4]; isolates from Amerindians either belonged to hpEurope or hspAmerind, and haplotypes from Mestizos were mostly hpEurope with a few hpAfrica1. We also included 19 hpAfrica1 strains from western Africa to reflect the African genetic influx to the Americas in colonial times, and 12 Korean strains (hspEAsia) to reflect the East Asian origins of Amerindians. In addition, we extracted the MLS sequences from 7 whole genomes available at the time of the analysis, including 4 from European hosts that were hpEurope (26695, HPAG1, G27, P12), one from a North American host that was hpAfrica1 (J99), and two from South American Native hosts that were hspAmerind (Shi470 and V225).

**Table 1:** ***H. pylori *haplotype as determined by MLS in 110 strains and by WGS in 7 strains, included in the *in silico *analysis**

**Host**	**Location**	**Ethnic group**	**N**	***H. pylori *haplotypes**			
				**hpAfrica1**	**hpEurope**	**hspEAsia**	**hspAmerind**
African (19)	Burkina Faso	Bantu	14	14			
	Senegal	Wolof	5	5			
European (14)	Italy	Italian	1		1*		
	Germany	German	1		1*		
	UK	English	1		1*		
	Sweden	Swedish	1		1*		
	Spain	Spanish	10		10		
Asian (12)	Japan	Japanese	1			1	
	Korea	Korean	11			11	
Native American (44)	Peru	Peruvian	1				1*
	Colombia	Huitoto	14		10		4
	Venezuela	Piaroa	7		2		5*
		Guahibo	3		3		
	Canada	Athabaskan	6				6
	Canada/ USA	Inuit	13		4		9
Mestizo (20)	Venezuela	Mestizo	9	4	5		
	Colombia	Mestizo	11	1	10		
North American (N = 1)	USA	North American	1	1*			
		**All**	**110**	**25**	**48**	**12**	**25**

*Whole genome sequence strain.

We determine the number of cognate recognition sites on the 110 MLS and 7 whole genome sequences (WGS) for 32 restriction/methylase enzymes previously reported in *H. pylori*. The number of cognate recognition sites per Kb on the 110 MLS and the 7 were highly consistent and comparable between the two types of sequences. To further validate that MLS are representative of the whole genome sequences, we performed a linear regression analysis. This analysis indicates a strong correlation between the observed cognate RMS sites frequencies in the 110 MLS and the seven WGS for the 32 RMS (Adjusted R^2^ = 0.80; p <0.001). Thus, MLS is representative of the whole genome sequences in terms of cognate RMS sites.

Of the 32 known cognate recognition sequences there were a mean (± SD) per Kb of 1.25 (± 1.26) in WGS and 1.55 (± 1.46) sites in MLS. In both WGS and MLS, the observed cognate recognition site frequencies were highly variable, ranging from 0 to 5.48 sites per Kb (Table 2). Although the distributions were relatively uniform (data not shown) along the DNA, there were several regions that showed coverage of <0.7 sites per Kb. Such sites often corresponded to "genomic islands" with G-C ratios (from 34.9% to 43.1% ± 4.1) that deviate from the intrinsic *H. pylori* ratio of about 39%. Expected recognition sites were calculated performing simulations on model sequences with the same length for the MLS and the WGS. These model sequences were constructed based on the average proportion of nucleotides of the actual sequences analyzed (Additional file 1: Table S1). To establish the expected frequencies of appearance of a specific recognition site by chance, we randomized the order of the nucleotides in the model sequences and enumerated the occurrence of that specific recognition site (see Methods for details). We estimated a range of 0.3 to 5.5 expected cognate recognition sites in both the MLS and WGS (R^2^ = 0.98, p < 0.001; Table 2). Overall, there were no significant differences in the observed or expected number of cognate restriction sites, among the haplotypes (p > 0.05).

**Table 2:** **Mean of the observed and expected combined values of the cognate recognition sites in *H. pylori *whole genome sequences and MLS for hspAmerind and hpEurope strains**

**RMS**		**Mean ± SD frequency/1.00 bp**				**O/E ratio**^**b**^	
**Endonuclease/ Methylase**	**Cognate recognition site**^**a**^	**MLS (N = 73)**		**WGS (N = 6)**			
		**Observed**	**Expected**	**Observed**	**Expected**	**MLS (N = 73)**	**WGS (N = 6)**
Hpy 166III	**CCTC**	2.7 ± 0.41	5.49 ± 0.07	2.93 ± 0.02	4.50 ± 0.03	**0.50**^**c**^	0.65
Hpy178VI	GGATG	1.48 ± 0.23	1.59 ± 0.03	0.81 ± 0.00	1.37 ± 0.01	0.93	0.59
Hpy17VII	GGCC	1.24 ± 0.31	1.96 ± 0.05	0.98 ± 0.02	1.43 ± 0.02	0.63	0.68
Hpy188I	**TCBGA**	1.02 ± 0.21	3.70 ± 0.03	0.81 ± 0.02	3.53 ± 0.01	**0.28**	**0.23**
Hpy188III	**TCBBGA**	1.11 ± 0.22	3.70 ± 0.04	1.19 ± 0.02	3.53 ± 0.01	**0.30**	0.34
Hpy8I	**GTNNAC**	0.40 ± 0.35	3.70 ± 0.03	0.22 ± 0.01	3.53 ± 0.01	**0.11**	**0.06**
Hpy8II	**GTSAC**	0.00 ± 0.00	1.56 ± 0.02	0.05 ± 0.00	1.37 ± 0.01	**0.00**	0.04
Hpy8III	GWGCWC	0.07 ± 0.12	0.66 ± 0.01	0.19 ± 0.01	0.19 ± 0.00	0.10	0.36
Hpy99I	CGWCG	0.28 ± 0.06	1.13 ± 0.02	0.15 ± 0.01	0.88 ± 0.01	0.25	0.17
Hpy99III	**GCGC**	4.62 ± 0.64	1.96 ± 0.05	3.73 ± 0.11	1.43 ± 0.02	**2.36**	**2.60**
Hpy99IV	CCNNGG	1.62 ± 0.26	1.96 ± 0.05	0.70 ± 0.01	1.43 ± 0.03	0.83	0.49
Hpy99VIP	GATC	5.48 ± 0.44	3.70 ± 0.03	3.19 ± 0.04	3.53 ± 0.01	1.48	0.90
Hpy99XIIP	**GTAC**	0.37 ± 0.20	3.70 ± 0.04	0.07 ± 0.00	3.53 ± 0.01	**0.10**	**0.02**
HpyAV	CCTTC(6/5)	0.58 ± 0.12	1.58 ± 0.02	0.80 ± 0.02	1.37 ± 0.01	0.37	0.58
HpyC1I	CCATC(4/5)	1.94 ± 0.26	1.94 ± 0.26	1.60 ± 0.02	1.39 ± 0.01	1.22	1.01
HpyCH4II	**CTNAG**	0.60 ± 0.28	3.70 ± 0.03	1.84 ± 0.04	3.53 ± 0.01	**0.16**	0.52
HpyCH4III	**ACNGT**	0.89 ± 0.22	3.70 ± 0.04	0.34 ± 0.00	3.53 ± 0.01	**0.24**	**0.10**
HpyCH4IV	**ACGT**	0.39 ± 0.22	3.70 ± 0.04	0.18 ± 0.01	3.53 ± 0.01	**0.11**	**0.05**
HpyCH4V	TGCA	3.85 ± 0.75	3.70 ± 0.03	3.45 ± 0.03	3.53 ± 0.03	1.04	0.98
HpyCI	GATATC	0.00 ± 0.03	0.31 ± 0.01	0.02 ± 0.00	0.33 ± 0.00	0.01	0.07
HpyF10VI	GCNNNNNNNGC	2.70 ± 0.35	1.96 ± 0.04	2.97 ± 0.09	1.43 ± 0.02	1.38	2.07
HpyF14I	CGCG	2.26 ± 0.46	1.96 ± 0.05	1.55 ± 0.05	1.43 ± 0.02	1.15	1.08
HpyF2I	CTRYG	1.16 ± 0.17	0.92 ± 0.01	0.37 ± 0.01	0.88 ± 0.00	1.26	0.42
HpyF36IV	GDGCHC	0.20 ± 0.21	1.22 ± 0.03	0.31 ± 0.01	0.93 ± 0.01	0.16	0.33
Hpy44II	GGNNCC	1.21 ± 0.38	1.96 ± 0.05	0.44 ± 0.00	1.43 ± 0.02	0.62	0.31
HpyII	GAAGA	2.29 ± 0.23	2.14 ± 0.03	2.87 ± 0.02	2.16 ± 0.00	1.07	1.33
HpyIP	CATG	4.63 ± 0.25	3.70 ± 0.03	4.43 ± 0.04	3.53 ± 0.01	1.25	1.25
HpyIV	GANTC	1.70 ± 0.25	3.70 ± 0.04	1.66 ± 0.02	3.53 ± 0.01	0.46	0.47
HpyNI	CCNGG	2.04 ± 0.30	1.96 ± 0.05	0.87 ± 0.02	1.43 ± 0.02	1.04	0.61
HpyPORF1389P	GAATTC	0.01 ± 0.05	0.31 ± 0.01	0.11 ± 0.00	0.33 ± 0.00	0.03	0.32
HpyV	**TCGA**	0.95 ± 0.25	3.70 ± 0.03	0.18 ± 0.00	3.53 ± 0.01	**0.26**	**0.05**
HpyVIII	CCGG	1.92 ± 0.30	1.96 ± 0.04	1.06 ± 0.02	1.43 ± 0.02	0.98	0.74

^a^Restriction endonucleases with palindromic recognition sites are indicated **in bold.** Code of degenerate nucleotide letters: R = G or A; Y = C or T; S = G or C; W = A or T; D = not C (A or G or T); H = not G (A or C or T); N = any nucleotide.

^b^O/E ratio indicates the observed/expected (O/E) ratio values. O/E ratios that are significantly (p-value <0.05) different from unity are highlighted **in bold** and bigger font.

^c^Exclusively underrepresented in hpEurope MLS.

The observed/expected (O/E) ratio indicates deviation from the expectation based on G + C ratio. O/E ratios were highly similar for the WGS and MLS (R^2^ = 0.87, p < 0.001), without any differences by haplotype. Analysis of the hpEurope and hspAmerind sequences showed that 10 of the 32 cognate restriction sites were underrepresented in MLS and 6 of those sites were also underrepresented in WGS (defined as O/E ≤ 0.5 and Chi Square p-value ≤ 0.005; Table 2). One exception, Hpy166III (cognate site: CCTC) was exclusively underrepresented in hpEurope MLS, but not in the hspAmerind nor in WGS. The underrepresented sites varied in their C + G content from 33.3 to 75%. Most (9) of those 10 underrepresented sites were palindromic [28-30] (Table 2). Conversely, only one cognate recognition site: Hpy99III (cognate site: GCGC), was strongly overrepresented (O/E ≥ 2 and Chi Square p-value ≤ 0.005) in both hpEurope/hspAmerind MLS and WGS (Table 2). Overall, similar results were found when analyzing hspEAsia and hspWAfrica strains (data not shown). In summary, the *H. pylori* genome has mostly evolved to avoid RMS cognate recognition sites. The total numbers of cognate restriction sites were not different among bacterial populations, based on *H. pylori* haplotypes.

### Profiles of cognate RMS recognition sites

The RMS profiles delineate the specific pattern of enzymatic recognition for each sequence, and offer more detailed information than the analysis of the total number of cognate recognition sites described above. Two strains with the same total number of cognate recognition sites among the combined pool of studied enzymes usually vary in the distribution of the specific cognate recognition sites for individual restriction enzymes within that pool. We found that the profile of RMS recognition sites varied significantly in a population-dependent manner (Wilcoxon rank sum test, p < 0.005). Four RMS sites (HPy99IV, HpyCH4V, HpyF14I, and HpyF44II) showed very strong directionality in the RMS strain profile, as shown by principal coordinate analysis (PCoA) of the 110 MLS (Additional file 1: Figure S2). Another 11 cognate recognition sites (Hpy166III, HpyNI, HpyC1I, Hpy8I, HpyIV, HpyF10VI, Hpy99VIP, HpyCH4II, Hpy188III, Hpy178VII, and HpyV) also contributed significantly, explaining 47% of the haplotype-strain variation (29% and 18%, respectively) amongst strains (Additional file 1: Figure S2). The other 17 recognition sites cumulatively explain only 9% of the total variation.

**Figure 1:**
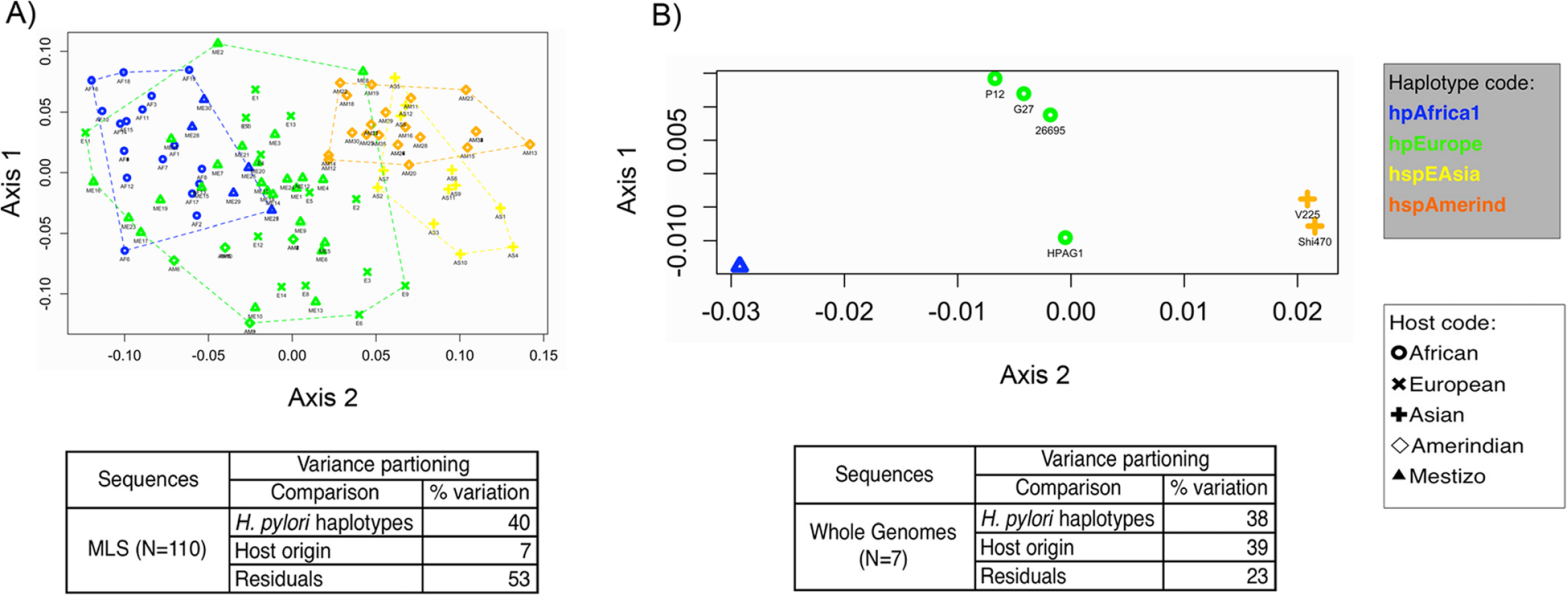
**Non-parametric multidimensional scaling (NMDS) based on the RMS profile for 15 restriction endonucleases in *H. pylori *DNA sequences.** NMDS is a visual representation of the most parsimonious distances, in terms of similarities and disparities, among the sequences. It provides a lower k-dimensional space, based on each restriction profile, which is the combination of the number of restriction sites for each of the 15 enzymes analyzed per sequence. Panel **A**: Analysis of 110 multilocus sequences. The restriction profile is distinct among haplotypes with the sequences clustering into groups, except for hpEurope that seems to have a more mixed restriction profile, with similarities with some hpAmerind and most hpAfrica1 strains. Panel **B**: Analysis of seven whole genome sequences. The restriction profile of the whole genome sequences is distinct among the *H. pylori* sub-groups, with hpEurope, hspAmerind, and hpAfrica1 clustering separated of each other.

Non-parametric multidimensional scaling (NMDS), based on those 15 cognate recognition site profiles that explain most of the variation in the PCA analyses also separated the *H. pylori* strains in a population-dependent way (Figure 1). Both for MLS and WGS analyses, the Amerindian and Asian strains exhibit similar profiles, that are distant from European and African strains that cluster apart (Adonis, p < 0.01). In contrast to the homogeneous African and Amerindian strains, the hpEurope strains from Mestizo or Amerindian hosts showed high heterogeneity in their restriction patterns (Figure 1). These results provide evidence for a phylogenetic signal in the profile of the frequencies of the cognate recognition sites in *H. pylori.*

**Figure 2:**
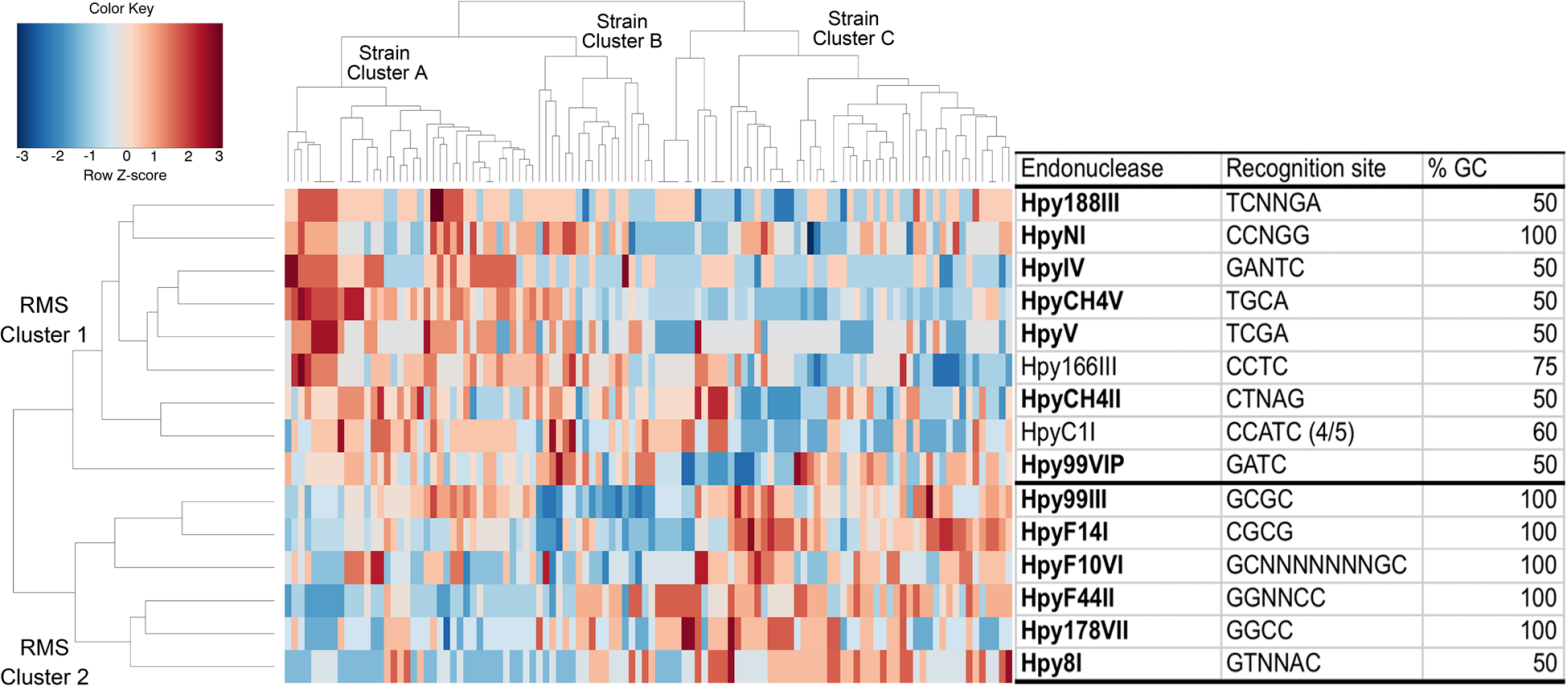
**Heatmap of the profile for 15 RE recognition sites on MLS DNA sequences for 110 *H. pylori *strains.** Higher and lower frequencies of the cognate recognition sites are represented by red and blue, respectively. The upper tree showed three main strainclusters: **A)** Includes hspAmerind (N=25), hspEAsia (N=5), and hpEurope (N=7) strains; **B)** Mostly hpEurope (N=21), but also hspEAsia (N=6), and hpAfrica1 (N=2) strains; and **C)** hpAfrica1 (N=23), and hpEurope (N=20) strains. The hpEurope strains studied were mostly recovered from Mestizo hosts. The phylogeny on the left shows two enzyme clusters, that correlate with the A, B and C cluster-strains.

A non-hierarchical analysis of the cognate recognition site profile for the same 15 RMS, with bidirectional clustering by frequency of the sites and by strain haplotype grouped RMS recognition sites (2 clusters), and strains (3 clusters, Figure 2). "Strain cluster A", with hspAmerind, hspEAsia and some hpEurope strains (from Amerindian and Mestizo hosts) has a high frequency of cognate sites for "RMS cluster 1". In contrast, the Euro-African "strain cluster C" has a low frequency of cognate sites for RMS in cluster 1, but high for "RMS cluster 2" (Figure 2). The cognate sites for RMS cluster 1 have a significantly lower G + C content compared to the cluster 2 cognate sites (59.4 ± 17.4 and 91.6 ± 20.4%, respectively. T-test = 0.002). "Strain cluster B" includes hspEAsia as well as hpEurope and hpAfrica1 from Mestizo and African hosts and shows a mosaic profile of the cognate recognition sites, consistent with the mosaic genetic structure shown in their MLS (Additional file 1: Figure S1).

### Strain-specific methylase representation

Differences in transformation rates might be due to differences in the frequency of cognate restriction sites, but also to variation in the protection conferred by active methylases belonging to the RMS. We tested the hypothesis that cognate restriction sites are more protected by active methylases in hpEurope than in hspAmerind strains. We selected 18 representative *H. pylori* strains; 9 were hpEurope recovered from European (n = 4), Mestizo (n = 4), and Amerindian (N = 1) hosts, and 9 were hspAmerind from Amerindian hosts (Additional file 1: Table S2). To determine methylase protection, genomic DNA from each strain was subject to digestion by each of 16 restriction endonucleases (Additional file 1: Table S3). Susceptibility to digestion indicated lack of an active methylase.

**Figure 3:**
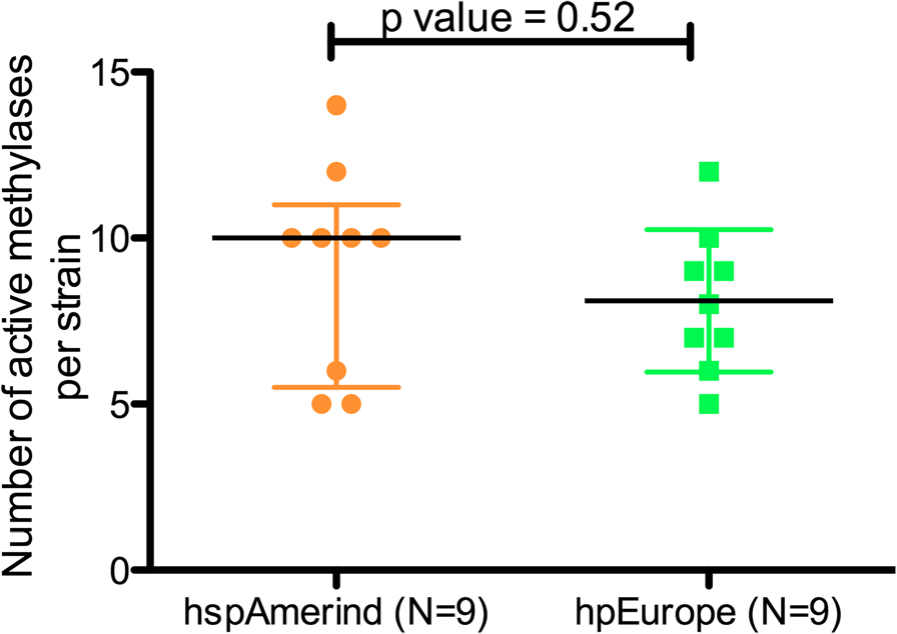
**Total number of active methylases per strain.** Both hspAmerind and hpEurope strains exhibited similar number of resistance to the 16 restriction enzymes tested.

The restriction results showed a range of 5–14 active methylases (average = 8.6 ± 2.6) per *H. pylori* strain of the 16 examined. There were non-significant differences in the number (Wilcoxon test, p > 0.05; Figure 3, Additional file 1: Table S3) or variances (F test, p > 0.05) of active methylases between hpEurope and hspAmerind strains. The only exception was the enzyme HpaII, to which DNA from the hspAmerind strains was significantly more resistant (83%) than DNA from the hpEurope strains (42%; Wilcoxon test; p < 0.05). Overall, the results confirm that *H. pylori* strains conserve similar active methylase protection, regardless of their population assignment.

### Genetic transformation rates

To assess differences in natural competence, five *H. pylori* hspAmerind strains isolated from Amerindians and five hpEurope strains recovered from European (N = 4) or Mestizo (N = 1) hosts each were transformed with two plasmids: i) p801R, a plasmid with an 800 bp insertion that introduces a single-base mutation of the gene *rpsL*, conferring resistance to Streptomycin (Str^R^); or ii) pCTB8, a plasmid with a 1.2 Kb insertion with an exogenous *aphA* cassette that produces Kanamycin-resistant (Km^R^) strains [31,32]. hspAmerind strains exhibited a significantly higher number of Str^R^ transformants than did hpEurope strains (3×10^-3^*vs*. 5×10^-5^, respectively; p < 0.005). Introduction of pCTB8 showed much lower rates of transformation: very few Kan^R^ colonies (1–3) were recovered, which did not allow comparison of the transformation frequency with this plasmid between the different *H. pylori* populations (data not shown).

We have hypothesized that the replacement of hspAmerind strains by hpEurope strains in Latin America was mainly facilitated by the introgression of DNA from hpEurope strains into hspAmerind strains [5]. To test this hypothesis, we reproduced the encounter of hspAmerind and hpEurope *H. pylori* strains by co-culturing and evaluating the directionality of the DNA horizontal transfers among strains *in vitro*. We produced double plasmid/resistant hspAmerind and hpEurope strains by transforming the single plasmid trains described above with an additional suicide plasmid, pAD1-Cat that includes an exogenous 1.3 Kb *cat* cassette that elicits Chloramphenicol resistance (Cm^R^). Thus, we obtained double resistant strains exhibiting: Str^R^/Cm^R^ or Km^R^/Cm^R^. To evaluate the direction of the DNA transformation, we co-cultured a single plasmid strain (used as the donor) with the double plasmid/resistant strain (as the recipient).

**Figure 4:**
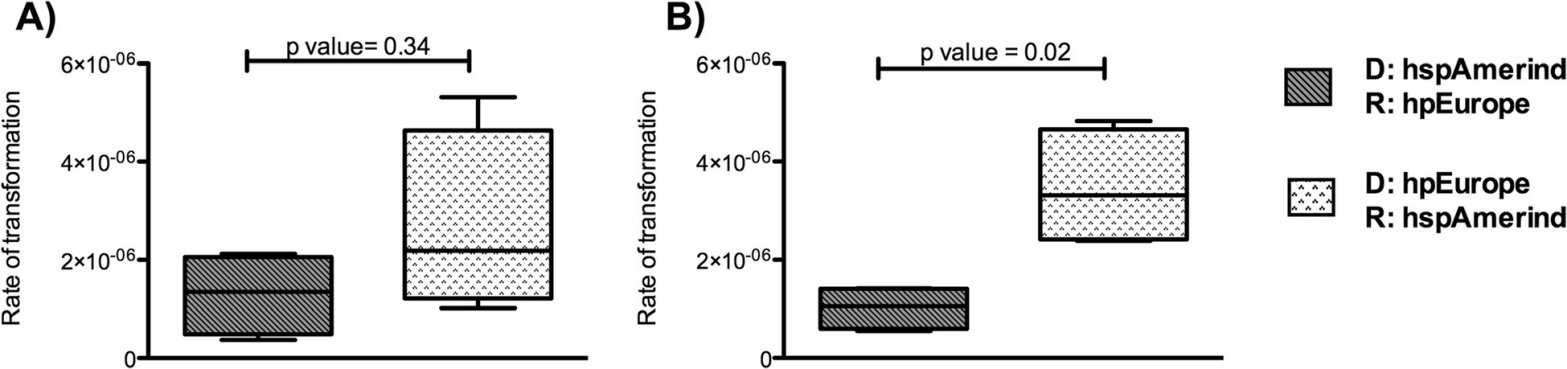
**Rate of transformation in different co-culture assays among hspAmerind and hpEurope strains.** The panel **A**, shows the rate of transformation of a single plasmid (p801R); in this case there was not significant differences when hspAmerind strains were donors (D) or recipients (R) of the DNA fragment. In the panel **B**, frequencies of transformation of a double plasmid (p801R+pAD1-*cat*) are showed. Amerindian strains exhibited higher ability to incorporate DNA from hpEurope than *vice versa*.

We first assessed the ability of *H. pylori* hspAmerind or hpEurope strains to acquire a plasmid with a single-base mutation (p801R) from each other, co-culturing Str^R^ strains (donor) and Cm^R^/Km^R^ strains (recipient). Transformants acquiring the single-base mutation from Str^R^ strains (p801R) will exhibit a triple antibiotic resistant phenotype: Str^R^/Cm^R^/Km^R^. The frequency of hspAmerind strains acquiring this single-base mutation from hpEurope strains was slightly higher (although not statistically significant, *p value* = 0.34) than hpEurope strains acquiring it from hspAmerind strains (Figure 4A). To extend our observation, we also co-cultured Str^R^/Cm^R^ and Km^R^ strains. We expected that during co-culturing, transformants acquiring the single-base mutation (p801R conferring Str^R^) from a Str^R^/Cm^R^ strain will be Str^R^/Km^R^ but Cm^S^, while transformants acquiring the 1.3 Kb *aphA* cassette from a Km^R^ strain will be triple antibiotic-resistant (Str^R^/Cm^R^/Km^R^). We observed that the frequency of transformation (1×10^-7^-1×10^-5^) with a single-base mutation (p801R), was higher than the frequency of transformants that had acquired the large DNA fragment (1.3 Kb pCBT8; <2×10^-8^) for both the hspAmerind and hpEurope strains. Control (blank) inoculations were included in all the transformation and co-culture experiments (see Methods) to control for spontaneous mutation events. The frequency of transformation of hspAmerind strains with the single-base mutation (Str^R^) from hpEurope (Str^R^/Cm^R^) strains was significantly higher (*p value* = 0.02) than that of hpEurope strains from hspAmerind strains (Figure 4B). For transformation events in which the 1.3 Kb *aphA* cassette is acquired from a Km^R^ strain (pCTB8), we observed that this cassette is not a suitable genetic marker to evaluate transformation between *H. pylori* strains because of the low frequency of transformation (<2 × 10^-8^); however, the few transform colonies (2–4 colonies per plate) were predominantly hspAmerind strains acquiring the cassette from hpEurope strains. In total, these observations support that Amerindian strains are more receptive to acquiring European DNA than *vice versa*.

## Discussion

### Phylogenetic signal of H. pylori RMS cognate sites and its correlation with human evolution

Our results confirm *H. pylori* genomic avoidance of many cognate restriction sites [33] In some bacteria, bacteriophages mimic the avoidance pattern of cognate recognition sites of their hosts [28,34-36] and exert selective pressure on the pattern of bacterial restriction sites [22,37]. Since bacteriophages do not appear important in *H. pylori*, presumably most of the pressure came from the RMSs themselves (22). Although we did not find significant haplotype differences in the frequencies of cognate recognition sites, we found population-specific differences in the profiles of the cognate recognition sites. The relatively more recent Asian and Amerindian *H. pylori* strains have lower frequencies of palindromic restriction sites rich in G + C than the African strains and also than the European strains which have been shown to be hybrids between an ancestral *H. pylori* population (ancestral Europe 1) from Central and Western Asia and another ancestral population (ancestral Europe 2) from Northeast Africa [1,2]. The genetic bottlenecks experienced by humans as they migrated from Africa [2,3], might also have influenced changes in the profile of frequency of restriction words in *H. pylori* strains. Indeed, the more homogeneous profile of restriction word frequencies in Amerindian *H. pylori* strains in relation to those from African and European strains (Figure 3), is consistent with the lower genetic diversity of both Amerindian hosts and their *H. pylori* strains [5].

What are the implications of this phylogenetic signature for the pattern of restriction site frequency in *H. pylori*? That G + C-rich restriction sites were both underrepresented and overrepresented, indicates a lack of selection for total G + C-content. Given that genetic drift is expected to be functionally neutral [2,4], we cannot discard that differences in the frequency of cognate restriction sites might be functionally relevant in *H. pylori*. This is consistent with the idea that RMS cognate recognition sites are important for recombination, an important force that drives the evolution of *H. pylori*. If modulation of natural competence occurs preferentially in one direction, this leads to genetic subversion of one of the transformed strains in a pair [18]. The results of this work suggest that the specific RMS cognate restriction site profile might lead to a recombination dynamic that favors "Europeanization" of Amerindian strains, explaining at least in part the replacement of Amerindian strains by European strains in Latin America.

In the context of human evolution, the human divergence within Africa and the worldwide divergence after the out-of-Africa migrations, were followed by genetic convergence by mixing in modern times. *H. pylori* strains differing in the use of cognate recognition words might have optimized fitness in the specific environment in which they evolved, but not in new host environments with different competitors. There may have been an ancestral *H. pylori* RMS pool, before out-of-Africa (around 60,000 years before present) followed by apparent differential selection for and avoidance of particular RMS, as *H. pylori* evolved with different isolated human groups. Selection against certain cognate recognition sites, particularly palindromes [26], has been shown in several bacteria and bacteriophages [38], which we again observe in *H. pylori*. The avoidance of specific palindromes may reflect selection pressure exerted by restriction enzymes with incomplete methylation [39], and their effects on genetic regulatory control [28,30]. When methylation protection fails, strains that avoid specific cognate restriction sites have a fitness advantage over those with more frequent cognate sites [30]. Consistent with this hypothesis is that life forms lacking RMS, such as some DNA viruses, mitochondria, and chloroplasts, do not show palindrome avoidance [29,30]. Differences in RMS profiles in the isolated sub-populations of *H. pylori* that derived from the worldwide spread of humans could reflect RMS competition, founder effects, and locale-specific selection.

The biological significance of overrepresentation of palindromic sites is harder to explain in the light of the defensive role of RMS. However, the frequent occurrence of small DNA fragments might increase recombination frequency, which may improve fitness [28,39]. Similarly, methylation of DNA promoters and origins of replication might provide benefits for the regulation of gene expression [40] and replication [41].

This study confirms prior observations that the mean numbers of active methylases are conserved in *H. pylori* strains recovered from hosts of different geographical origins [42,43], suggesting selection for an optimal RMS number across the universe of *H. pylori* cells [42,44]. Such selection might be achieved by horizontal gene transfer of RMS genes among *H. pylori* strains, with a consequent equilibrium in the number of active methylases. RMSs have been postulated to behave as "selfish" mobile genetic elements [27,45,46]. Selection favors the maintenance of the system of restriction endonuclease and methylase, because loss of methylase function is lethal. However, intact methylase genes with apparently truncated restriction genes have been observed in completed *H. pylori* genomes, suggesting that active methylases are involved in the regulation of essential physiological processes that are independent of RMS [47]. However, the process of restriction and methylation might be a dynamic mechanism that can vary *in vivo*. For example, HpyI methylase (HpyIM) expression varied dramatically within *H. pylori* cells colonizing the gastric tissue [48].

### Dominance of European over Amerindian strains

Despite a similar number of active methylases, hspAmerind strains exhibited higher rates of transformation than hpEurope strains. DNA incorporation into the chromosome during transformation can be divided into three general steps: i) DNA uptake or binding to the cell; ii) degradation of one strand of the invading DNA, and iii) recombination of the remnant DNA fragments into the genome [49,50]. For the first step, extensive evidence supports the fact that *H. pylori* is highly competent in uptake of "non-self" DNA. *H. pylori* is genetically diverse within a single stomach niche and is subject to a very high rate of intraspecific recombination [11,14,51]. Proteins such as ComB4, ComB7–ComB10 of the type IV secretion system encoded by the *comB* genes, [52] are homologs to VirB proteins (VirB4, VirB7–VirB10) of *A. tumefaciens* and resemble their conjugation-like function in *H. pylori* DNA transformation [53]. Mutations of *comB* in *H. pylori* strains abrogate transformation [52,54]. Whether haplotype differences in the proteins involved in DNA uptake and access to foreign DNA can affect the efficiency of DNA uptake and incorporation, remains to be tested. Step (ii) involves the degradation of one DNA strand and processing of the foreign DNA. Although *H. pylori* isolates from different bacterial populations exhibit a similar number of methylases, the differences in the cognate recognition sites can explain differences in the "DNA availability" as a substrate for recombination. For example, four-base cognate recognition sites are (~16-fold) more frequent that six-base recognition sites. Step (iii), homologous recombination, requires at least a single stranded break; DNA differences in the location of the homologous sites may favor higher transformation in Amerindian strains. When two *H. pylori* strains meet in a host’s stomach, they can recombine in an asymmetric fashion, leading to subversion of one strain by the other.

An additional explanation of European dominance might rely on host selection that seems to favor European strains, for example, host mixing with Europeans. Host selection is evidenced by the *H. pylori* adhesin phenotypes in relation to human blood groups. Up to 95% of "generalist" European *H. pylori* strains can bind A, B or O antigens whereas 60% of Amerindian strains bind only O antigens [55]. This binding-specialization of *H. pylori* strains coincides with the unique predominance of blood group O antigens in Amerindian hosts. Our results provide evidence that asymmetric recombination rates lead to dominance of one strain over another by means of genetic subversion. If Amerindian strains recombine at higher rates, they are more likely to become mosaic strains integrating European loci and gradually become "Europeanized".

## Conclusions

**Figure 5:**
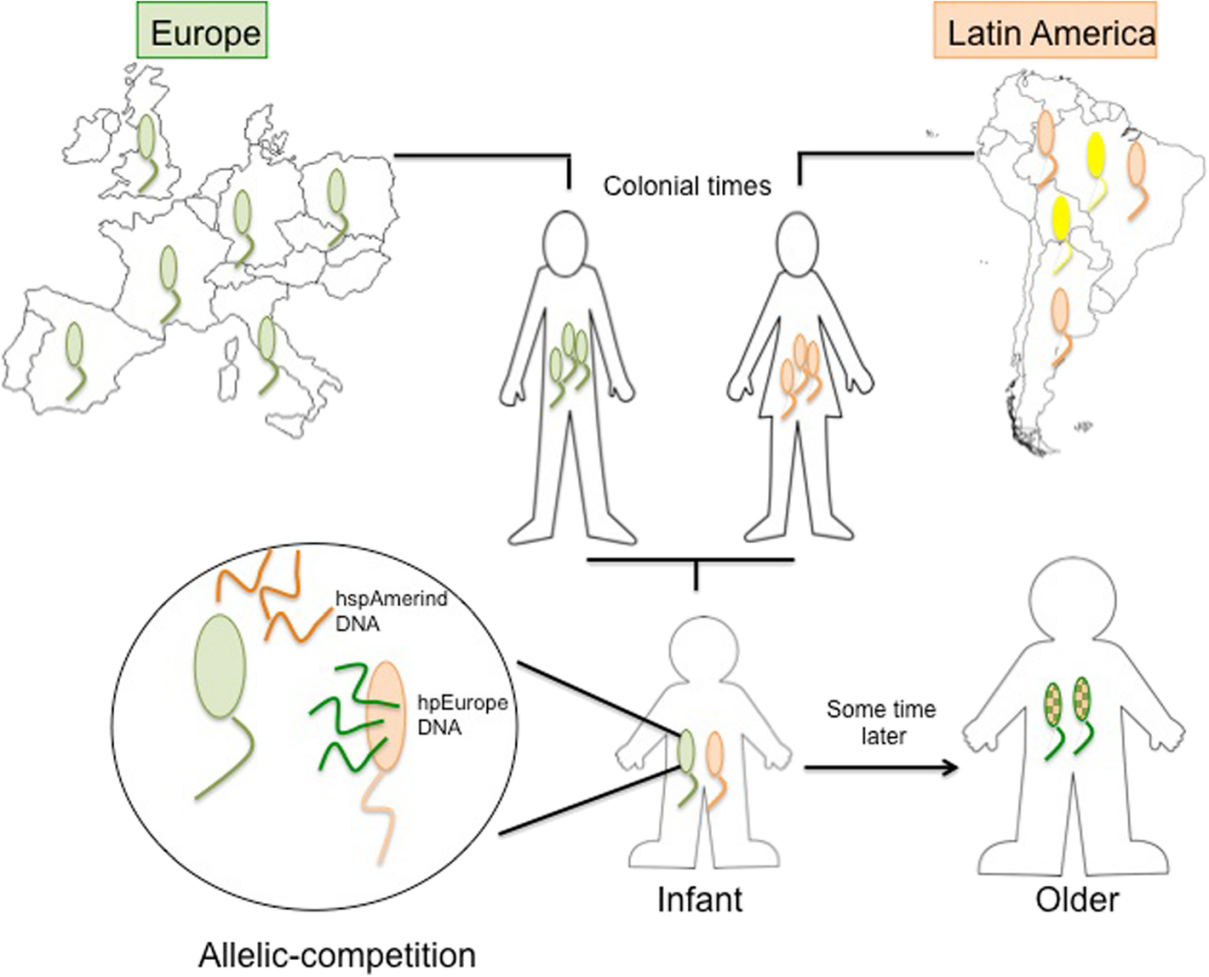
**Model of *H. pylori *strain dynamics in Latin America hosts.** The different color of the bacteria (green and orange) represents the MLS profile and the cognate restriction profile of *H. pylori* strains. Ancestral strains from Europe and Latin America Amerindians share common genetic signature, both MLS [1,2] and cognate restriction profile (as shown in our results). In colonial times where European and Amerindians mixed, we hypothesize that the new generation will acquire *H. pylori* from both parents. Within a single host (mestizos) allelic competition will occurs among strains and hpEurope DNA take over hspAmerind strains promoting its Europeanization (demonstrated in our co-culture results) and mosaic genetic structure.

In conclusion, geographical variations in the pattern of cognate recognition sites provide evidence for ancestral differences in RMS representation and possibly also in function. The higher transformation rates in Amerindian strains support the hypothesis of Europeanization of Latin American strains via recombination. A potential scenario, supported by our results is that during colonial times when Spanish conquers, African slaves, and Native Amerindians mix also did their *H. pylori* haplotypes, thus a new generation of *H. pylori* strains arise, exhibiting mosaic genetic structure result of several events of recombination among strains with different RMS profile. In this mixing, hpEurope alleles succeed dominating their incorporation into DNA from Amerindian strains (See Figure 5). Future studies are needed to evaluate differences by haplotype in competence-related function driven by *comB, dprA* and *comH* genes [56,57].

## Methods

### *In silico* analysis

#### Sequences

We analyzed 117 DNA sequences of *H. pylori* strains, 110 of which were partial sequences and seven were whole genomes. The partial sequences were a string of 3,406 bp composed of ordered concatenated sequences (multilocus sequences, or MLS) from seven housekeeping genes as follows: *atpA* (627 bp)*, efp* (410 bp)*, mutY* (420 bp)*, ppa* (398 bp)*, trpC* (456 bp)*, ureI* (585 bp) and *yphC* (510 bp) [58-60]*.* The MLS were from *H. pylori* strains from hosts from four continents: Africa, Europe, Asia, and the Americas (from Native American and Mestizo hosts). All sequences were available at the EMBL or GenBank database (http://www.ebi.ac.uk/) and/or at the MLST website for *H. pylori* (http://pubmlst.org/helicobacter/) [59]. Whole genome sequences (WGS ~ 1.5 Mb) of seven *H. pylori* were available in GenBank. Four strains were from European hosts: 26695, HPAG1, P12 and G27 (accession numbers NC_000915, NC_008086, NC_ 011333, CP001173, respectively; all hpEurope); one, J99 (NC_000921; hpAfrica1) was from the US, and two Shi470 and V225 (NC_010698; CP001582; hspAmerind) were from Native Americans from Peru and Venezuela, respectively. The MLS of the 7 strains with whole genome sequences were also taken into account for the analysis, and form part of the 110 MLS analyzed.

#### Haplotype assignment

All the sequences were previously analyzed and assigned to their correspondent populations [2,5]. Neighbor joining clustering analysis [61] of all the strains was performed in MEGA 5.0. [62].

#### Frequency of cognate recognition sites

The observed frequency of cognate recognition sites for 32 RMS (Table 2) that have been reported in *H. pylori*[25,42,43,63] was determined in the 110 MLS (3,406 bp) and 7 WGS (1.5-1.7 Mb) using the EMBOSS restriction program (http://emboss.sourceforge.net/), by counting the number of restriction "words", in each sequence. We determined: 1) the number of cognate recognition sites, that is the sum of all words per strain, 2) their frequency per Kb, 2) their distribution per Kb in the seven WGS, and 4) the RMS profile of each strain, which is the combination of the values for the 32 cognate recognition sites per strain. The expected frequency of cognate recognition sites was based on the actual nucleotide proportions in each WGS or MLS sequence (Additional file 1: Table S2), and determined by 1,000 simulations. The algorithm used for simulating the frequencies of cognate recognition sites was created as follows: (i) a pool of 1,000 nucleotides containing the exact proportion of each nucleotide in each genome or MLS sequence was created (the "pool-simulated sequence"); (ii) a nucleotide was randomly chosen, from the pool-simulated sequence, *k* times, in which *k* is the length of each recognition sequence; (iii) simulated words that matched the recognition sequence were counted; and steps 2, 3 were repeated *l-k* times, where *l* is the length of the whole genome or MLS sequence.

For each enzyme, observed and expected numbers of cognate recognition sites were compared (O/E ratio) values per enzyme. We estimated the Chi square of the observed and expected values of the cognate recognition sites per haplotype. Underrepresentation was defined when the O/E ratio value was lower than 0.5, and the Chi square value was significant (p values <0.005). Similarly, the sites were overrepresented in the sequences when the ratio O/E value was ≥2, and the Chi square value was significant (p values <0.005). In the case of WGS, we calculated Chi square only for the bacterial populations that contained more than one strain: hpEurope (26695, HPAG1, P12 and G27), and hspAmerind (V225 and Shi470), but not for hpAfrica1 with just one strain (J99). Differences in the frequency of observed and expected cognate recognition sites among *H. pylori* populations were examined using a pair-wise comparison test based on the medians (Wilcoxon rank sum test). For the 4 populations studied (hspWAfrica, hpEurope, hspEAsia, and hspAmerind), there were 6 possible pair-wise analyses. The p-value for the Wilcoxon rank sum test for each pair indicates the relationships among the haplotypes. Principal component analysis (PCoA) [64] was performed to detect patterns of cognate recognition profiles among strains. Non-parametric multidimensional scaling (NMDS), was used to visualize the variation in two dimensions [65]. NMDS does not assume linearity of the data and does not require data transformation, which represents advantages over other classical ordination methods. The ordination algorithm for NMDS clusters groups with similarities, and based on ranked similarity distances; an iterative search for the least stress position in *k*-dimensions is done [65].

### *In vitro* analysis

#### Bacterial strains for restriction analysis

Nine hspAmerind strains from Amerindian hosts (N = 9), and nine hpEurope strains from European (N = 4) and Mestizo (N = 5) hosts were used for this analysis. The 18 frozen cultures of *H. pylori* strains, maintained at -80°C, were thawed and inoculated onto Brucella agar plates supplemented with 5% blood [66]. Plates were incubated at 37°C in a microaerobic atmosphere (5% CO_2_) in a humid chamber for 3 to 5 days [66]. *H. pylori* identity was confirmed by Gram staining and detection of urease and catalase activity. DNA was extracted from *H. pylori* cultures using the Wizard® Genomic DNA Purification Kit (Promega, MA), with the protocol specified by the manufacturer for gram-negative bacteria.

#### Restriction assays

Restriction endonuclease digestions were performed on the genomic DNA from 18 strains, using 16 commercially available restriction enzymes (New England BioLabs, MA) that were sensitive to methylation of the recognition sites (Additional file 1: Table S3). These enzymes were chosen because resistance to each has been reported in at least one *H. pylori* strain [42]. In our experiments, we controlled for the lack of restriction activity due to presence of inhibitors or high salt, by running control DNA from an *H. pylori* strain with a known restriction profile [18,42]. However, in addition to the possibility of lacking the cognate restriction sites, lack of restriction activity due to the presence of supercoiled DNA cannot be ruled out.

In the restriction assays, ~500 μg of DNA were digested with 5U of the specified endonucleases for 2 h in a final volume of 30 μl of the appropriate buffer as recommended by the manufacturer. Chromosomal DNA from *E. coli* DH5α, as well as the *H. pylori* strains HPK5 and 99–35, were used as positive controls, to assess activity of the enzymes. Digestion products were electrophoresed at 80 V for 1 h in a 1% agarose gel [42]. The number of active methylases was determined based on the sensitivity of the DNA to restriction. The variable responses to the independent digestions were dichotomous: (lack of digestion) presence of the active methylase = 1 or 0 = digestion, no active methylase. To examine the differences in the number of active methylases between the bacterial populations, Wilcoxon-sum rank test was performed*.*

#### Transformation analysis

*H. pylori* hspAmerind or hpEurope strains with Str^R^, or Km^R^ genetic markers were obtained by transformation with plasmid p801R or pCBT8, as described [32] and listed in Table 3. Plasmid p801R contains *rspL* with a point mutation in position 128 (A128G substitution), which confers resistance to Streptomycin (Str^R^). Plasmid pCTB8 carries an *aphA* cassette, which is integrated into the genome on the transformation-unrelated *vacA* locus and confers Kanamycin resistance (Km^R^).

**Table 3:** **Plasmids and *H. pylori *mutant strains used in the co-colonization studies**

**Plasmids and code strains**		**Relevant characteristics**	**Source or reference**
Suicide plasmids	p801R	pGEM-T easy, *H. pylori* 26695 *rpsL* fragment with A128G point mutation	(Levine *et al.*, 2007)
	pCTB8	pGEM-T easy, *H. pylori vacA::aphA*	(Cover *et al.*, 1994)
	pAD1-Cat	pGEM-T easy, *H. pylori ureA::cat*	(Lin *et al.*, 2001)
*H. pylori* strains	99-33	hspAmerind	(Takata *et al.*, 2002)
	99-35	hspAmerind	(Takata *et al.*, 2002)
	08-97	hpEurope	This study
	08-100	hpEurope	This study
	99-33 + p801R	hspAmerind/ Str^R^	This study
	99-35 + p801R	hspAmerind/ Str^R^	This study
	08-97 + p801R	hpEurope/ Str^R^	This study
	08-100 + p801R	hpEurope/ Str^R^	This study
	99-33 + pCTB8	hspAmerind/ Km^R^	This study
	99-35 + pCTB8	hspAmerind/ Km^R^	This study
	08-97+ pCTB8	hpEurope/ Km^R^	This study
	08-100 + pCTB8	hpEurope/ Km^R^	This study
	99-33 + p801R + pAD1-Cat	hspAmerind/ Str^R^/Cm^R^	This study
	99-35 + p801R + pAD1-Cat	hspAmerind/ Str^R^/Cm^R^	This study
	08-97 + p801R + pAD1-Cat	hpEurope/ Str^R^/Cm^R^	This study
	08-100 + p801R + pAD1-Cat	hpEurope/ Str^R^/Cm^R^	This study
	99-33 + pCTB8+ pAD1-Cat	hspAmerind/ Km^R^/Cm^R^	This study
	99-35 + pCTB8+ pAD1-Cat	hspAmerind/ Km^R^/Cm^R^	This study
	08-97 + pCTB8+ pAD1-Cat	hpEurope/ Km^R^/Cm^R^	This study
	08-100 + pCTB8+ pAD1-Cat	hpEurope/ Km^R^/Cm^R^	This study

In each case, the transformants can be detected based on the resistance phenotype of the transformed cells onto selective media. In brief, *H. pylori* strains were inoculated and incubated at 37°C in 5% CO_2_[31] for 3 days. The cells were re-plated and re-incubated for 24–72 h under the same conditions, then re-suspended in 1000 μl of PBS using a sterile swab and then centrifuged at 1,500 g for 2 min. The pellet was re-suspended in 200 μl of PBS, 25 μl of the *H. pylori* cells were mixed with 15 μl of the plasmid at a final concentration of 30 ng/μl. The mix was plated on Brucella agar supplemented with 5% sheep blood (BAB) and incubated as described above. After 24 h, the colonies were collected with a sterile swab and diluted in series from 10^-1^ to 10^-6^ in 900 μl Brucella broth (BB). The first four dilutions were spotted on selective media: BAB + Str [20 μg/mL], or Km [10 μg/mL], depending of the phenotype to be selected. The two last dilutions were inoculated onto non-selective BAB plates. After 5 days of incubation, colony-forming units (CFU) were counted on both the selective and non-selective plates, and transformation efficiency was calculated by comparing CFU numbers on the two types of media.

CFU counts used for this analysis were over a range of 30 – 300, to maximize statistical accuracy [67]. Differences in the rates of transformation were compared using the t-test, and the variance among strains was determined using the F-test.

#### Horizontal DNA transfer during co-culture

To evaluate the ability of *H. pylori* hspAmerind or hpEurope strains to obtain DNA from each other, the co-culture assay was performed as previously described [32]. The strains and plasmids used for these experiments are listed in Table 3. In summary, in addition to the single plasmid strains explained above, we produced double-resistant hspAmerind and hpEurope strains by transforming the single resistant strains described above with an additional suicide plasmid, pAD1-Cat [32]. This suicide plasmid, which carries a *ure*AB fragment from *H. pylori* strain 60190 with a central exogenous *cat* cassette (1127 bp), gets incorporated into the genomic *ureA* locus, creating chloramphenicol resistant (Cm^R^) strains [32].

To determine the rates of DNA transformation from a donor hspAmerind strain to a recipient hpEurope strain, a single plasmid hspAmerind strain (99–33 or 99–35) with resistance to antibiotic "X" (used as a donor) and a double plasmid hpEurope strain (08–97 or 08–100) with resistance to antibiotics "Y/Z" (used as recipient), were co-cultured; transformants were selected by double or triple antibiotic resistance: "X/Y" or "X/Y/Z", respectively. To investigate the rates of transformation from a donor hpEurope strain to a recipient hspAmerind strain, we performed the same experiment but with the reverse phenotype, i.e. donor = hpEurope with single resistance "X"; recipient = hspAmerind with double resistance "Y/Z", and transformants with double or triple antibiotic resistance: X/Y" or "X/Y/Z", were evaluated. Based on the observation that after co-culturing a Str^R^/Cm^R^ strain and a Km^R^ strain, the transformants with Str^R^/Km^R^ are substantially higher (>2 log_10_ CFU) than those with Str^R^/Cm^R^/Km^R^, we concluded that the rate of transformation with Str^R^/Km^R^ could be used to reflect the Km^R^ strain acquiring the single-base mutation *rpsL* (Str^R^) from the Str^R^/Cm^R^ strain.

To test for spontaneous mutations, blank controls we included in co-culture experiments, with recipient strains (i.e. Str^R^/Cm^R^ resistant) plated in selective plates containing the antibiotic for the donor strains (i.e. Str^R^). Resistant strains due to spontaneous mutations were never observed. As described above, results were based on CFU counts. Comparisons among the rates of transformation obtained from hspAmerind and hpEurope strains were assessed by performing the Mann Whitney test. For all transformation experiments, we used the appropriate blank controls for selection. Non-transformed strains were subject to the same conditions and plated on non-selective media to confirm cell viability.

## Competing interests

All the authors declare that they have no competing interests.

## Authors’ contributions

ALM designed the analysis, perform all the *in silico* analysis, restriction and transformation experiments, analyzed the data and perform statistics, also prepared the manuscript and figures. MS optimized the mathematical model for expected restriction sites and perform all the simulation analysis. XZ perform the co-culture experiments and participate in the manuscript preparation. PL help with the initial statistical modeling for the simulation analysis. AT and MC provided samples to the completion of the study. LB help analyzing MLST to be assigned to specific haplotype, also collaborate in the manuscript preparation. MGDB and MB participate in the experimental design, discussion of results, preparation and review of the manuscript. All authors read and approved the final manuscript.

## Supplementary Material

Additional file 1: Table S1 Proportion of nucleotides in the *H. pylori* sequences analyzed. **Table S2**. Haplotype and origin of the strains included in the *in vitro* analysis of active methylases. **Table S3**. Distribution of active methylases in *H. pylori* strains, by haplotype. **Figure S1**. Neighbor joining clustering based on multilocus sequences of 110 *H. pylori* strains used in this study*.* The strains were grouped (Kimura-2 parameter) into four main clusters accordingly with the population assignment using STRUCTURE software: hpAfrica1 (N=25) in blue, hpEurope (N=48) in green; hspEAsia (N=12) in yellow and hspAmerind (N=25) in orange. **Figure S2.** PCA showing the variation among *H. pylori* strains. PCA is a mathematical model that transforms the data to a new coordinate system. The data is organized based on coordinates that goes from the one with the greatest variance by any projection (called the first principal component), to the second greatest variance on the second coordinate, and so on. Based on the frequency of cognate recognition sites for 32 endonucleases, *H. pylori* strains were separated in two coordinates. Strains are coded by haplotype: **AM** for hspAmerind, **AS** for hspEAsia, **E** for hpEurope, and **AF** for hpAfrica1. The number that follow the haplotype code indicate the sequence number (e.g. hspAmerind, N=25= AM1, AM2… AM25). Zero (0) indicates no variation. Arrows in red indicate the direction of the variation for each of the 32 restriction sites analyzed; longer arrows indicate that the variation of the restriction profile for a given, is far from zero (more variable). Differences in the RMS profile were mainly due to 15 cognate recognition sites for: HpyCH4V, HpyF14I, Hpy99IV, Hpy166III, HpyF44II, HpyNI, HpyC1I, Hpy8I, HpyIV, HpyF10VI, Hpy99VIP, HpyCH4II, Hpy188III, Hpy178VII, HpyV endonucleases; which explained 29% and 18% of the variation in component 1 and 2, respectively. Click here for file
