# Correction: Phylogeographic evidence of cognate recognition site patterns and transformation efficiency differences in *H. pylori*: theory of strain dominance

**DOI:** 10.1186/1471-2180-14-122

**Published:** 2014-05-12

**Authors:** Ana Maldonado-Contreras, Shrinivasrao P Mane, Xue-Song Zhang, Luis Pericchi, Teresa Alarcón, Monica Contreras, Bodo Linz, Martin J Blaser, María Gloria Domínguez-Bello

**Affiliations:** 1 Department of Biology, University of Puerto Rico, Río Piedras, San Juan, PR, USA; 2 Microbiology and Physiological Systems Department, University of Massachusetts Medical School, Worcester, MA, USA; 3 Virginia Bioinformatics Institute, Virginia Tech, Blacksburg, VA, USA; 4 Department of Medicine, New York University Langone Medical Center, Manhattan, NY, USA; 5 Department of Mathematic, University of Puerto Rico, Río Piedras, San Juan, USA; 6 Servicio de Microbiología, Hospital Universitario de la Princesa, Madrid, Spain; 7 Venezuelan Institute of Scientific Research (IVIC), San Antonio de los Altos, Venezuela; 8 Department of Biochemistry and Molecular Biology, Pennsylvania State University, University Park, PA, USA; 9 New York Harbor Veterans Affairs Medical Center, Manhattan, NY, USA

## Correction

In this published article [1], a couple of typos in Table 2 were found:

•The restriction enzymes Hy17VII and the Hpy44II should be Hpy178II and HpyF44II instead.

•The cognate restriction site for Hpy188I, Hpy188III and HpyF2I should be TCNGA, TCNNGA, and CTRYAG, respectively.

•The subtitle ‘Mean ± SD the frequency’ should by followed by ‘/1,000 bp’ instead of ‘/1.00 bp’

•The footnotes were misleading and are now corrected.

In addition, we apologize for the omission of following source that it should be included on the methods section as a reference:

Roberts RJ, Vincze T, Posfai J, Macelis D (2010) REBASE--a database for DNA restriction and modification: enzymes, genes and genomes. Nucl. Acids Res. 38: D234D236.

**Table 2:** **Mean of the observed and expected combined values of the cognate recognition sites in *H. pylori *** **whole genome sequences and MLS for hspAmerind and hpEurope strains**

**RMS**		**Mean ± SD frequency/1,000 bp**				**O/E ratio **^**b**^	
**Endonuclease/ Methylase**	**Cognate recognition site **^**a**^	**Observed**	**Expected**	**Observed**	**Expected**	**MLS (N = 73)**	**WGS (N = 6)**
Hpy166III	**CCTC**	2.7 + 0.41	5.49 + 0.07	2.93 + 0.02	4.50 + 0.03	**0.50 **^**c**^	0.65
Hpy178VI	GGATG	1.48 + 0.23	1.59 + 0.03	0.81 + 0.00	1.37 + 0.01	0.93	0.59
Hpy178VII	GGCC	1.24 + 0.31	1.96 + 0.05	0.98 + 0.02	1.43 + 0.02	0.63	0.68
Hpy188I	**TCNGA**	1.02 + 0.21	3.70 + 0.03	0.81 + 0.02	3.53 + 0.01	**0.28**	**0.23**
Hpy188III	**TCNNGA**	1.11 + 0.22	3.70 + 0.04	1.19 + 0.02	3.53 + 0.01	**0.30**	0.34
Hpy8I	**GTNNAC**	0.40 + 0.35	3.70 + 0.03	0.22 + 0.01	3.53 + 0.01	**0.11**	**0.06**
Hpy8II	**GTSAC**	0.00 + 0.00	1.56 + 0.02	0.05 + 0.00	1.37 + 0.01	**0.00**	0.04
Hpy8III	GWGCWC	0.07+ 0.12	0.66 + 0.01	0.19 + 0.01	0.54 + 0.00	0.10	0.36
Hpy99I	CGWCG	0.28 + 0.06	1.13 + 0.02	0.15 + 0.01	0.88 + 0.01	0.25	0.17
Hpy99III	**GCGC**	4.62 + 0.64	1.96 + 0.05	3.73 + 0.11	1.43 + 0.02	**2.36**	**2.60**
Hpy99IV	CCNNGG	1.62 + 0.26	1.96 + 0.05	0.70 + 0.01	1.43 + 0.03	0.83	0.49
Hpy99VIP	GATC	5.48 + 0.44	3.70 + 0.03	3.19 + 0.04	3.53 + 0.01	1.48	0.90
Hpy99XIIP	**GTAC**	0.37 + 0.20	3.70 + 0.04	0.07 + 0.00	3.53 + 0.01	**0.10**	**0.02**
HpyAV	CCTTC (6/5)	0.58 + 0.12	1.58 + 0.02	0.80 + 0.02	1.37 + 0.01	0.37	0.58
HpyC1I	CCATC(4/5)	1.94 + 0.26	1.60 + 0.02	1.39 + 0.01	1.37 + 0.01	1.22	1.01
HpyCH4II	**CTNAG**	0.60 + 0.28	3.70 + 0.03	1.84 + 0.04	3.53 + 0.01	**0.16**	0.52
HpyCH4III	**ACNGT**	0.89 + 0.22	3.70 + 0.04	0.34 + 0.00	3.53 + 0.01	**0.24**	**0.10**
HpyCH4IV	**ACGT**	0.39 + 0.22	3.70 + 0.04	0.18 + 0.01	3.53 + 0.01	**0.11**	**0.05**
HpyCH4V	TGCA	3.85 + 0.75	3.70 + 0.03	3.45 + 0.03	3.53 + 0.03	1.04	0.98
HpyCI	GATATC	0.00 + 0.03	0.31 + 0.01	0.02 + 0.00	0.33 + 0.00	0.01	0.07
HpyF10VI	GCNNNNNNNGC	2.70 + 0.35	1.96 + 0.04	2.97 + 0.09	1.43 + 0.02	1.38	2.07
HpyF14I	CGCG	2.26 + 0.46	1.96 + 0.05	1.55 + 0.05	1.43 + 0.02	1.15	1.08
HpyF2I	CTRYAG	1.16 + 0.17	0.92 + 0.01	0.37 + 0.01	0.88 + 0.00	1.26	0.42
HpyF36IV	GDGCHC	0.20 + 0.21	1.22 + 0.03	0.31 + 0.01	0.93 + 0.01	0.16	0.33
HpyF44II	GGNNCC	1.21 + 0.38	1.96 + 0.05	0.44 + 0.00	1.43 + 0.02	0.62	0.31
HpyII	GAAGA	2.29 + 0.23	2.14 + 0.03	2.87 + 0.02	2.16 + 0.00	1.07	1.33
HpyIP	CATG	4.63 + 0.25	3.70 + 0.03	4.43 + 0.04	3.53 + 0.01	1.25	1.25
HpyIV	GANTC	1.70 + 0.25	3.70 + 0.04	1.66 + 0.02	3.53 + 0.01	0.46	0.47
HpyNI	CCNGG	2.04 + 0.30	1.96 + 0.05	0.87 + 0.02	1.43 + 0.02	1.04	0.61
HpyPORF1389P	GAATTC	0.01 + 0.05	0.31 + 0.01	0.11 + 0.00	0.33 + 0.00	0.03	0.32
HpyV	**TCGA**	0.95 + 0.25	3.70 + 0.03	0.18 + 0.00	3.53 + 0.01	**0.26**	**0.05**
HpyVIII	CCGG	1.92 + 0.30	1.96 + 0.04	1.06 + 0.02	1.43 + 0.02	0.98	0.74

^a^ Code of degenerate nucleotide letters: R = G or A; Y = C or T; S = G or C; W = A or T; D = not C (A or G or T); H = not G (A or C or T); N = any nucleotide.

^b^ O/E ratio indicates the observed/expected (O/E) ratio values. O/E ratios that are significantly (pvalue <0.05) different from unity are highlighted **in bold**.

^c^ Exclusively underrepresented in hp Europe MLS.
