# Supplementary material for: Phylogeographic evidence of cognate recognition site patterns and transformation efficiency differences in H. pylori: theory of strain dominance
Source: BMC Microbiol. 2013 Sep 19;13:211. doi: 10.1186/1471-2180-13-211 (PMC3849833; doi:10.1186/1471-2180-13-211)
Supplement: Additional file 1: Table S1 — Proportion of nucleotides in the H. pylori sequences analyzed. Table S2. Haplotype and origin of the strains included in the in vitro analysis of active methylases. Table S3. Distribution of active methylases in H. pylori strains, by haplotype. Figure S1. Neighbor joining clustering based on multilocus sequences of 110 H. pylori strains used in this study. The strains were grouped (Kimura-2 parameter) into four main clusters accordingly with the population assignment using STRUCTURE software: hpAfrica1 (N=25) in blue, hpEurope (N=48) in green; hspEAsia (N=12) in yellow and hspAmerind (N=25) in orange. Figure S2. PCA showing the variation among H. pylori strains. PCA is a mathematical model that transforms the data to a new coordinate system. The data is organized based on coordinates that goes from the one with the greatest variance by any projection (called the first principal component), to the second greatest variance on the second coordinate, and so on. Based on the frequency of cognate recognition sites for 32 endonucleases, H. pylori strains were separated in two coordinates. Strains are coded by haplotype: AM for hspAmerind, AS for hspEAsia, E for hpEurope, and AF for hpAfrica1. The number that follow the haplotype code indicate the sequence number (e.g. hspAmerind, N=25= AM1, AM2… AM25). Zero (0) indicates no variation. Arrows in red indicate the direction of the variation for each of the 32 restriction sites analyzed; longer arrows indicate that the variation of the restriction profile for a given, is far from zero (more variable). Differences in the RMS profile were mainly due to 15 cognate recognition sites for: HpyCH4V, HpyF14I, Hpy99IV, Hpy166III, HpyF44II, HpyNI, HpyC1I, Hpy8I, HpyIV, HpyF10VI, Hpy99VIP, HpyCH4II, Hpy188III, Hpy178VII, HpyV endonucleases; which explained 29% and 18% of the variation in component 1 and 2, respectively. [file 1471-2180-13-211-S1.pdf]

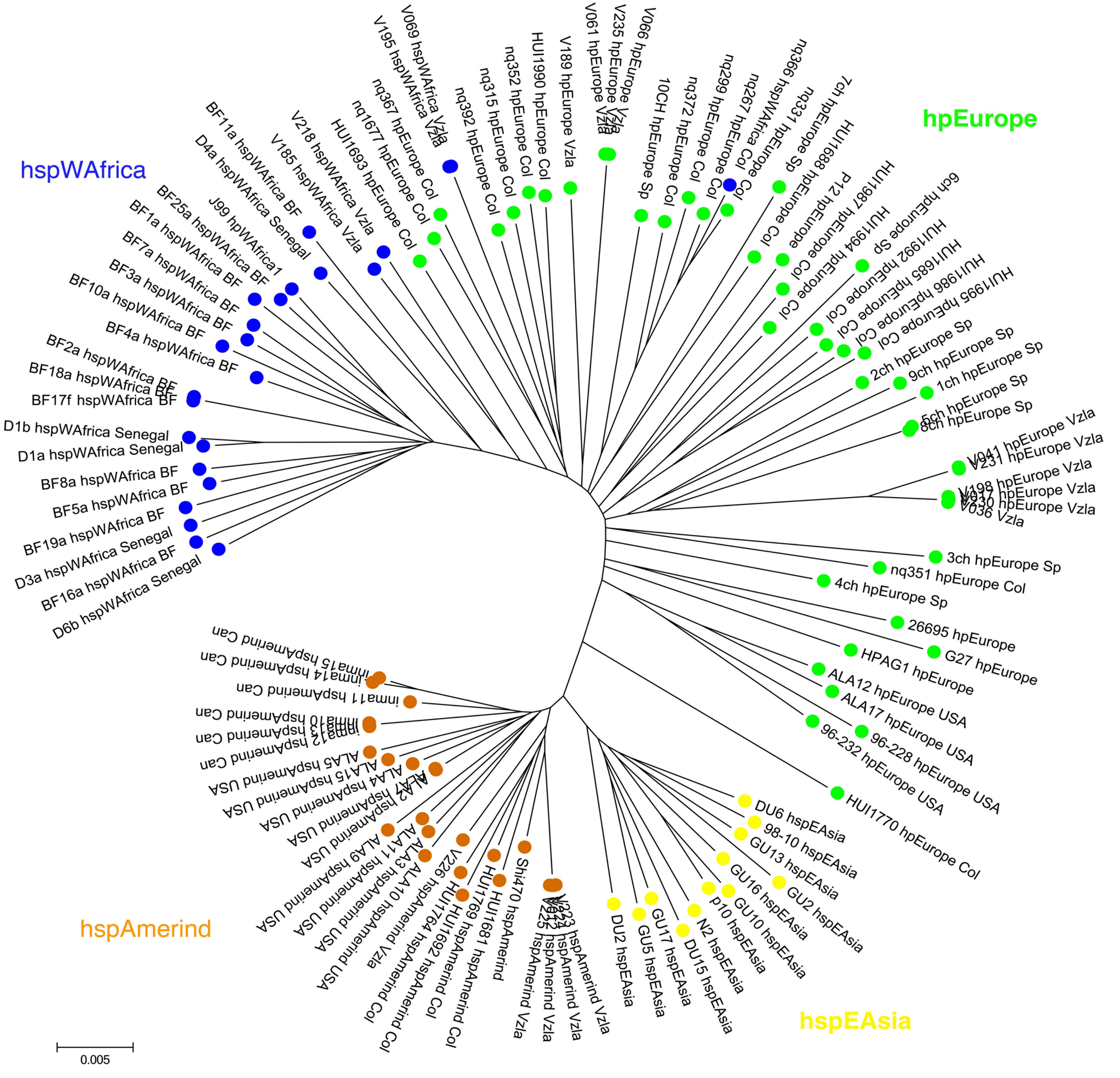

hspWAfrica
hpEurope
hspAmerind
hspEAsia
BF11a hspWAfrica BF
D4a hspWAfrica Senegal
J99 hpWAfrica1
BF25a hspWAfrica BF
BF1a hspWAfrica BF
BF7a hspWAfrica BF
BF3a hspWAfrica BF
BF10a hspWAfrica BF
BF4a hspWAfrica BF
BF2a hspWAfrica BF
BF18a hspWAfrica BF
BF17f hspWAfrica BF
D1b hspWAfrica Senegal
D1a hspWAfrica Senegal
BF8a hspWAfrica BF
BF5a hspWAfrica BF
BF19a hspWAfrica BF
D3a hspWAfrica Senegal
BF16a hspWAfrica BF
D6b hspWAfrica Senegal
V185 hspWAfrica Vzla
V218 hspWAfrica Vzla
HUI1693 hpEurope Col
nq1677 hpEurope Col
nq367 hpEurope Col
V195 hspWAfrica Vzla
V069 hspWAfrica Vzla
nq392 hpEurope Col
nq315 hpEurope Col
nq352 hpEurope Col
HUI1990 hpEurope Col
V189 hpEurope Vzla
V061 hpEurope Vzla
V235 hpEurope Vzla
V066 hpEurope Vzla
10CH hpEurope Sp
nq372 hpEurope Col
nq299 hpEurope Col
nq267 hpEurope Col
nq366 hspWAfrica Col
nq331 hpEurope Sp
7ch hpEurope Sp
HUI1688 hpEurope Col
P12 hpEurope
HUI1987 hpEurope Col
6ch hpEurope Sp
HUI1994 hpEurope Col
HUI1992 hpEurope Col
HUI1685 hpEurope Col
HUI1986 hpEurope Col
HUI1995 hpEurope Col
2ch hpEurope Sp
9ch hpEurope Sp
1ch hpEurope Sp
5ch hpEurope Sp
8ch hpEurope Sp
V041 hpEurope Vzla
V231 hpEurope Vzla
V198 hpEurope Vzla
V017 hpEurope Vzla
V030 hpEurope Vzla
V036 Vzla
3ch hpEurope Sp
nq351 hpEurope Col
4ch hpEurope Sp
26695 hpEurope
G27 hpEurope
HPAG1 hpEurope
ALA12 hpEurope USA
ALA17 hpEurope USA
96-228 hpEurope USA
96-232 hpEurope USA
HUI1770 hpEurope Col
DU6 hspEAsia
98-10 hspEAsia
GU13 hspEAsia
GU2 hspEAsia
GU16 hspEAsia
GU10 hspEAsia
p10 hspEAsia
N2 hspEAsia
DU15 hspEAsia
GU17 hspEAsia
GU5 hspEAsia
DU2 hspEAsia
V223 hspAmerind Vzla
V225 hspAmerind Vzla
Shi470 hspAmerind
HUI1681 hspAmerind
HUI1769 hspAmerind Col
HUI1692 hspAmerind Col
HUI1764 hspAmerind Col
V226 hspAmerind Vzla
ALA10 hspAmerind USA
ALA3 hspAmerind USA
ALA11 hspAmerind USA
ALA9 hspAmerind USA
ALA2 hspAmerind USA
ALA4 hspAmerind USA
ALA15 hspAmerind USA
ALA5 hspAmerind USA
inma12 hspAmerind Can
inma13 hspAmerind Can
inma10 hspAmerind Can
inma11 hspAmerind Can
inma14 hspAmerind Can
inma15 hspAmerind Can
0.005

Figure S1

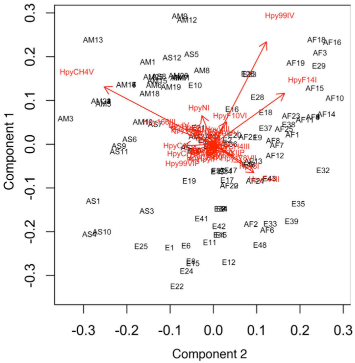

Component 1
Component 2
0.3
0.2
0.1
0.0
-0.1
-0.2
-0.3
Hpy99IV
HpyCH4V
HpyF14I
HpyNI
HpyF10VI
Hpy99VIP
AM9
AM12
AM13
AM1
AS12
AS5
AM8
AM19
AM18
AM3
AM15
AM6
E10
AF18
AF16
AF3
AF19
E29
AF15
AF10
AF14
E28
E16
E18
AF23
E38
E37
AF25
AF1
AF8
AF7
AF12
E32
AS6
AS9
AS11
AS7
E19
E17
AF24
E43
AS1
AS3
AS10
AS4
E35
E39
E33
AF2
AF6
E41
E42
E11
E48
E25
E1
E6
E8
E15
E24
E12
E22

Figure S2

TABLE S1. Proportion of nucleotides in the *H. pylori* sequences analyzed.

Nucleotide	% in Whole Genomes (N=7)[a]	% in MLS (N=110)[b]
T	30.6 ± 0.1	29.1 ± 0.2
C	19.6 ± 0.2	19.5 ± 0.2
A	30.4 ± 0.1	28.6 ± 0.2
G	19.4 ± 0.1	22.7 ± 0.2

[a] Between 1,254,207 and 1,673,813 nucleotides were examined per whole genome.
[b] A total of 3,406 nucleotides were examined per MLS.

TABLE S2. Haplotype and origin of the strains included in the *in vitro* analysis of active methylases.

Strain code	Country-host ethnicity	*H. pylori* haplotype	
		hspAmerind	hpEurope
AM1	Venezuela-Piaroa	X	
AM2	Venezuela-Piaroa	X	
AM3	Venezuela-Piaroa	X	
AM4	USA-Inuit	X	
AM5	USA-Inuit	X	
AM6	USA-Inuit	X	
AM7	Colombia-Huitoto	X	
AM8	USA-Inuit	X	
AM9	USA-Inuit	X	
AM10	USA-Inuit	X	
AM11	Venezuela-Piaroa	X	
AM12	Peru-Amerindian	X	
SP1	Spain-Spanish		X
SP2	Spain-Spanish		X
SP3	Spain-Spanish		X
SP4	Spain-Spanish		X
MEST1	Peru-Mestizo		X
MEST2	Peru-Mestizo		X
MEST3	Venezuela-Mestizo		X
MEST4	Peru-Mestizo		X
MEST5	Colombia-Mestizo		X
MEST6	Colombia-Mestizo		X
MEST7	Venezuela-Mestizo		X
MEST8	Venezuela-Mestizo		X

TABLE S3. Distribution of active methylases in *H. pylori* strains, by haplotype

Restriction Enzyme (RE)	Cognate recognition site	% of strains resistant to specific RE	
		H. pylori population	
		hspAmerind (N=9)	hpEurope (N=9)
DdeI	CTNAG	22	33
FokI	GGATG	11	11
HaeIII	WGGCCW	56	56
HinfI	GANTC	44	56
HpaII	GTTAAC	89	44
Hpy188I	TCNGA	56	44
Hpy188III	TCNNGA	33	44
Hpy99I	CGWCG	56	67
HpyCH4III	ACNGT	56	22
HpyCH4IV	ACGT	100	78
HpyCH4V	TGCA	56	56
MboI	GATC	100	100
MboII	GAAGA	44	44
MwoI	GCNNNNNNNGC	11	0
NlaIII	CATG	100	89
TaqI	TCGA	78	56
Mean $\pm$ SD		9 $\pm$ 3	9 $\pm$ 2
Range		(5 - 15)	(5 - 12)
